# Supplementary material for: Evaluating miR-107 and adiponectin as biomarkers in obstructive sleep apnea: Associations with neurotransmitters and metabolic regulation
Source: PLoS One. 2025 May 5;20(5):e0322128. doi: 10.1371/journal.pone.0322128 (PMC12052186; doi:10.1371/journal.pone.0322128)
Supplement: Attachment — Submitted filename: recommendation.docx [file pone.0322128.s003.docx]

Experiments have been conducted rigorously, with appropriate controls and replication. Sample sizes large enough to produce robust results. Methods and reagents are described in sufficient detail for another researcher to reproduce the experiments described.

The data presented in the manuscript support the conclusions drawn. The interpretation of results is justified and appropriate. Authors have discussed possible implications for their results. The article is presented in an intelligible fashion and is written in Standard English (clear, correct, and unambiguous).The article adheres to appropriate reporting guidelines and community standards for data availability. Results are appropriately reported.
