# Supplementary material for: Evaluating miR-107 and adiponectin as biomarkers in obstructive sleep apnea: Associations with neurotransmitters and metabolic regulation
Source: PLoS One. 2025 May 5;20(5):e0322128. doi: 10.1371/journal.pone.0322128 (PMC12052186; doi:10.1371/journal.pone.0322128)
Supplement: Attachment — Submitted filename: Response to Reviewers.docx [file pone.0322128.s005.docx]

**Phakkharawat Sittiprapaporn
Academic Editor
PLOS ONE**

Dear Phakkharawat,

We would like to express our sincere gratitude for the opportunity to submit our manuscript titled " **Evaluating miR-107 and adiponectin as biomarkers in obstructive sleep apnea: Associations with neurotransmitters and metabolic regulation**" to PLOS ONE. We deeply appreciate the insightful comments and suggestions provided by you and the reviewers, which have greatly contributed to improving our work.

We have carefully considered each comment and made the necessary revisions to the manuscript. Below is our point-by-point response to the reviewers' comments:

**Reviewer #1:**

**Comment 1: Comparison of miR-107 and Neurotransmitters Across Different Groups**

We appreciate the reviewer’s insightful comments and suggestions. In response, we have conducted the requested analyses across the specified groups. The results of these analyses have been compiled in the supplementary file titled "Detailed Analysis." This file provides a comprehensive summary, including mean, standard deviation (SD), skewness, kurtosis, Shapiro-Wilk test statistics with corresponding p-values, Spearman's correlation coefficients with p-values, and Mann-Whitney U test statistics with p-values.

Our findings indicate that no statistically significant correlations were observed between miR-107 and other markers across all groups, except for significant correlations between miR-107 and serotonin in the male control group, and between miR-107 and glutamate in the female control group. Additionally, significant distribution differences were observed among all groups for all studied markers.

It is important to note that the initial analysis did not include these group-wise comparisons due to the limited number of participants in certain subgroups, particularly in the postmenopausal control group, as well as to some extent in the male and premenopausal groups. However, the overall sample size for the case and control groups was statistically sufficient to draw meaningful conclusions.

For future studies, we recommend ensuring adequate representation across all subgroups to facilitate more robust subgroup analyses. The primary objective of our study was to assess the feasibility of using these biomarkers for the diagnosis of OSA. We acknowledge this limitation and appreciate the reviewer’s valuable feedback, which has provided us with further insights to improve both the current study and our future research endeavors.

Thank you once again for your constructive input.

**Comment 2: Manuscript Language and Clarity**

We sincerely appreciate the reviewer’s valuable feedback and constructive suggestions. In response, we have thoroughly revised the entire manuscript, paying close attention to the issues highlighted. Additionally, we carefully reviewed the text to identify and correct any other potential errors. We are grateful for the reviewer’s efforts, which have significantly contributed to enhancing the quality and value of our article.

**Reviewer #2:**

We sincerely thank the reviewer for their positive feedback and appreciation of our work. We are pleased to know that our experimental rigor, methodological clarity, and data interpretation meet the required standards. Your encouraging comments motivate us to further uphold the quality and reproducibility of our research. We are grateful for your time and effort in reviewing our manuscript.

**Reviewer #3:**

**Comment 1: Clarification of miR-107 Detection Method Using ELISA**

We appreciate the reviewer’s concern. Due to the unavailability of qRT-PCR facilities at the time of our study, we opted for the ELISA method as a reliable and validated alternative for miR-107 quantification. The detection was performed using the Human miR-107 ELISA Kit (Shanghai Ideal Medical Technology, China), strictly following the manufacturer’s protocol. Below is the detailed procedure as outlined in the accompanying pamphlet of the kit.

''Human miR-107 ELISA Kit employs the sandwich enzyme immunoassay technique for the quantitative measurement of human miR-107 in serum, plasma, tissue homogenates, and other biological fluids. An antibody specific for miR-107 has been pre-coated onto a 96-well microtiter plate. The standards and test samples are added into the wells and the miR-107 present in each sample is bound to the wells by the immobilized antibody. Following incubation, the wells are washed and then incubated with Biotinylated Anti-miR-107 Antibody, which binds the captured miR-107 present in each well. Following incubation, unbound biotinylated detection antibody is removed by washing, and an HRP-Streptavidin conjugate is added to the wells and the microtiter plate is incubated. Following incubation and washing, TMB substrate solution is then used to visualize the HRP enzymatic reaction by catalysis to produce a blue-colored product that changes to yellow after addition of acidic stop solution. The density of yellow is proportional to the amount of miR-107 captured in each well. The concentration of miR-107 can then be calculated by reading the O.D. absorbance at 450nm in a microplate reader and referring to the standard curve.''

**Comment 2: Consistency in miR-107 Notation**

We appreciate the reviewer’s observation regarding the inconsistency in the notation of miR-107. In response, we have carefully reviewed the manuscript and ensured that the notation has been standardized to "miR-107" throughout the text. Thank you for bringing this to our attention.

**Comment 3: Clarification of Study Conclusions**

We thank the reviewer for pointing out this concern. We acknowledge the unintentional overstatement in the abstract and have revised the concluding sentence to better align with the study’s findings. We have ensured that our conclusions accurately reflect the scope and implications of our results. We appreciate the reviewer’s valuable feedback in improving the clarity and precision of our manuscript.

**Comment 4: Correlation Analysis Between miR-107 and Biomarkers**

We appreciate the reviewer’s comment and would like to clarify that Spearman's correlation analysis between miR-107, and the studied biomarkers has been conducted and is presented in the results section of the manuscript. Our analysis of the control samples revealed statistically significant moderate correlations between miR-107 and certain biomarkers, while no significant correlations were observed with the other studied markers. We have ensured that the significance and implications of these findings are appropriately discussed in the manuscript.

Thank you for your valuable feedback.

**Comment 5: Improvement of Logical Flow in Introduction and Justification for miR-107 Selection**

We appreciate the reviewer’s suggestion to enhance the logical flow of the Introduction. In response, we have restructured the section to prioritize a discussion on existing biomarkers for OSA and their limitations, followed by an explanation for the selection of miR-107.

Different biomarkers have been explored to assess the presence and severity of OSA and to evaluate its systemic effects. While biomarkers such as C-reactive protein (CRP), interleukins (IL-6, IL-8), and tumor necrosis factor-alpha (TNF-α) reflect the systemic inflammation associated with OSA and its comorbidities, they lack specificity for OSA. Similarly, oxidative stress biomarkers, including malondialdehyde (MDA), 8-isoprostane, and oxidized LDL, are associated with intermittent hypoxia and reoxygenation but suffer from a lack of standardization. Genetic biomarkers, such as TNF-α gene polymorphisms and angiotensin-converting enzyme gene polymorphisms, have been suggested to predict susceptibility to OSA or its complications. However, the complex polygenic nature of OSA, coupled with genetic variability and environmental influences, makes it challenging to identify a single definitive genetic marker. As a result, no single genetic marker is likely to provide conclusive diagnostic or prognostic information.

Given these limitations, current research is focused on identifying more precise, reliable biomarkers for OSA to complement polysomnography, the gold standard for diagnosis. In this context, miR-107 was selected as a promising biomarker due to its non-invasive nature, specificity, and direct link to the molecular events triggered by intermittent hypoxia. miR-107 has shown a strong correlation with the arousal index and sleep parameters, which are crucial in OSA. Furthermore, miR-107 regulates key pathways involving glutamate, serotonin, adiponectin, and melatonin, all of which influence cognitive function, metabolic health, and respiratory control. Dysregulation of miR-107 exacerbates glutamate toxicity, serotonin imbalance, reduced adiponectin, and disrupted melatonin production, all of which are implicated in OSA pathology.

The decision to focus on miR-107 was driven by its potential to address these interconnected issues. While further research is necessary to overcome current limitations and confirm its diagnostic and prognostic value, miR-107 stands out as one of the most promising biomarkers for advancing the clinical management of OSA.

This revision aims to enhance the logical progression of the Introduction by first addressing existing biomarkers, their limitations, and then justifying the selection of miR-107 based on its potential to overcome these challenges.

**Comment 6: Correction of Figure 1**

Thank you for your feedback. We included the table as Fig 1 in accordance with the journal's guidelines, which require tables presented in figure format to be referred to as figures rather than tables.

**Comment 7: Discussion of BMI Differences and Influence of Obesity on miR-107 and Adiponectin**

We appreciate the reviewer's insightful comments and observations. In our study, miR-107 and adiponectin levels were found to be lower in OSA patients compared to controls. The observed reductions in miR-107 and adiponectin are likely influenced by both obesity and OSA. Obesity is known to induce a state of low-grade chronic inflammation and elevate the production of pro-inflammatory cytokines, which can suppress miR-107 expression and inhibit adiponectin secretion. Furthermore, adiponectin levels are typically reduced in obese individuals, irrespective of OSA status.

In addition to obesity, OSA-specific pathophysiological mechanisms, such as intermittent hypoxia, contribute to systemic inflammation and oxidative stress, which may further downregulate miR-107 expression and impair adiponectin secretion. The recurrent episodes of airway obstruction and consequent hypoxemia exacerbate metabolic dysregulation in OSA patients, further influencing these biomarker levels.

While obesity is a significant contributing factor to these alterations, OSA-related mechanisms, including hypoxia and inflammation, also play a critical role. Ideally, a case group comprising non-obese OSA patients would provide a clearer understanding of the independent effects of OSA on these biomarkers. However, the recruitment of such individuals presents a considerable challenge, as non-obese OSA patients are relatively uncommon in clinical practice.

To mitigate the confounding effects of obesity and isolate the specific impact of OSA, we selected non-obese individuals as controls. This approach allows for a more precise evaluation of OSA-related biomarker changes, such as those driven by intermittent hypoxia and systemic inflammation, without the additional influence of obesity.

We acknowledge the reviewer’s concern regarding the selection of the control group; however, our approach was based on the need to delineate OSA-related effects from those associated with obesity, while also considering the practical limitations in recruiting non-obese OSA patients.

**Final words:**

We would like to inform the editor that the data used for this study has been uploaded as supporting files. We greatly appreciate your time and effort in considering our manuscript, and we would like to thank you for your valuable feedback and support throughout the review process.

Kind regards,

Dr. Kaleem Arshad

Khawaja M. Safdar Medical College, Sialkot

[kaleemarshad630@gmail.com](mailto:kaleemarshad630@gmail.com)
