# Supplementary material for: Evaluating miR-107 and adiponectin as biomarkers in obstructive sleep apnea: Associations with neurotransmitters and metabolic regulation
Source: PLoS One. 2025 May 5;20(5):e0322128. doi: 10.1371/journal.pone.0322128 (PMC12052186; doi:10.1371/journal.pone.0322128)
Supplement: Attachment — Submitted filename: Response_to_Reviewers_auresp_2.docx [file pone.0322128.s006.docx]

**Phakkharawat Sittiprapaporn
Academic Editor
PLOS ONE**

Dear Dr. Phakkharawat,

We would like to express our sincere gratitude for the opportunity to submit our manuscript titled “**Evaluating miR-107 and adiponectin as biomarkers in obstructive sleep apnea: Associations with neurotransmitters and metabolic regulation**” to PLOS ONE. We deeply appreciate the insightful comments provided by you and the reviewers.

We received two comments in total. One reviewer recommended the manuscript for publication, and the only point requiring our response was regarding the miR-107 estimation. As there were no concerns or suggestions for modifications to the manuscript text, no changes to the manuscript were necessary.

Regarding the reviewer’s comment on miR-107 measurement: We clarified that the miR-107 quantification was performed using the Human miR-107 ELISA Kit, strictly following the manufacturer’s instructions. Both cases and controls were assayed concurrently, and miR-107 levels were determined by comparing the optical density at 450 nm against a standard curve generated from the kit-provided standards. We are confident that this approach ensured accurate and reproducible measurements, as further supported by our simultaneous quantification of other markers using the same instruments and laboratory conditions.

**Reviewer #1:**

Dear Reviewer, Thank you for your insightful comment. We would like to clarify that the quantification of miR-107 was performed using the Human miR-107 ELISA Kit, and the procedure was strictly carried out according to the manufacturer's manual. Specifically, the assay employs a sandwich enzyme immunoassay technique for the quantitative measurement of human miR-107 in serum, plasma, tissue homogenates, and other biological fluids. The full method is as follows:

“Human miR-107 ELISA Kit employs the sandwich enzyme immunoassay technique for the quantitative measurement of human miR-107 in serum, plasma, tissue homogenates, and other biological fluids. An antibody specific for miR-107 has been pre-coated onto a 96-well microtiter plate. The standards and test samples are added into the wells and the miR-107 present in each sample is bound to the wells by the immobilized antibody. Following incubation, the wells are washed and then incubated with Biotinylated Anti-miR-107 Antibody, which binds the captured miR-107 present in each well. Following incubation, unbound biotinylated detection antibody is removed by washing, and an HRP-Streptavidin conjugate is added to the wells and the microtiter plate is incubated. Following incubation and washing, TMB substrate solution is then used to visualize the HRP enzymatic reaction by catalysis to produce a blue-colored product that changes to yellow after addition of acidic stop solution. The density of yellow is proportional to the amount of miR-107 captured in each well. The concentration of miR-107 can then be calculated by reading the O.D. absorbance at 450nm in a microplate reader and referring to the standard curve. Both cases and controls were assayed simultaneously using the same batch of reagents, instruments, and standardized protocols, ensuring minimal inter-assay variability. We also quantified other biomarkers in parallel using the same instrumentation and laboratory conditions, and those measurements confirmed the overall consistency and reliability of our procedures.”

While we acknowledge that our results indicate a wide difference in miR-107 levels between OSA patients and controls, literature does report significant differences in miRNA expression across groups, albeit not always as pronounced as in our study. It is important to note that at the time of our study, we faced constraints including limited access to a qRT-PCR facility and budget restrictions. Consequently, we opted for the ELISA method, which was already in use for the quantification of other markers in our laboratory. Moreover, as the participants were recruited last year, the available serum samples have been fully utilized, and the current funding does not allow for additional qRT-PCR analyses.

We are confident in the accuracy and validity of our results, as the procedures were rigorously standardized and executed, ensuring reliable data. We appreciate your valuable feedback and hope this response adequately addresses your concerns.

**Reviewer #4:**

Thank you very much for your positive feedback and for recommending our manuscript for publication. We truly appreciate the time and effort you invested in reviewing our work. Your constructive comments and recognition of the enhancements made to the clarity and robustness of our study have been invaluable to us. We are delighted that the modifications have satisfactorily addressed the concerns raised and look forward to the next steps.

**Final Words:**

Thank you again for the opportunity to submit our work and for your valuable feedback. We hope our responses have adequately addressed the comments and look forward to your favorable decision on the acceptance of our manuscript.

Kind regards,

Dr. Kaleem Arshad

Khawaja M. Safdar Medical College, Sialkot

[kaleemarshad630@gmail.com](mailto:kaleemarshad630@gmail.com)
